# Current status and influencing factors of policy identification in health impact assessment: a case study of Zhejiang Province

**DOI:** 10.1186/s12961-023-01064-9

**Published:** 2023-11-06

**Authors:** Xiang Liu, Yingzi Liu, Yanyun Xu, Liyuan Song, Ziyue Huang, Xingyu Zhu, Meng Zhang

**Affiliations:** School of Public Health, Hangzhou Normal University, 2318 Yuhangtang Road, Hangzhou, 311121 China

**Keywords:** China, Health impact assessment, Policy identification

## Abstract

**Background:**

Health impact assessment (HIA) is a procedure, method and tool for evaluating the potential health impacts of policies, plans and construction projects, as well as the distribution of these impacts on population. Majority of international studies on health impact assessment have focussed on conceptual papers or case evaluations, neglecting participants’ views on policies.

**Methods:**

A semi-structured interview with 30 health impact assessment experts was employed in this study, and the Nvivo software was utilized to analyse factors that influence policy identification. Subsequently, a multi-stage stratified random sampling method was adopted to survey 655 pilot staff members involved in health impact assessment in Zhejiang Province. Descriptive statistics were used to describe the current status and identify the factors influencing policy identification. In addition, hierarchical linear regression analysis and structural equation modelling were employed to determine the relationship between policy identification and influencing factors.

**Results:**

Statistically significant differences were found among participants in the level of identification of policies across three dimensions. The policy sentiment dimension had the highest score (4.137 ± 0.664), followed by policy cognition (4.075 ± 0.632) and policy evaluation (3.631 ± 0.797) dimensions. Subject trust had a positive impact on policy cognition (*β* = 0.503, *P* < 0.001), policy sentiment (*β* = 0.504, *P* < 0.001) and policy evaluation (*β* = 0.465, *P* < 0.001). Procedural justice had a positive impact on policy sentiment (*β* = 0.085, *P* < 0.01) and policy evaluation (*β* = 0.084, *P* < 0.05), but not policy cognition (*β* = 0.056, *P* > 0.05). Policy identification is influenced by age and average monthly salary among other factors.

**Conclusion:**

These results highlight the importance of subjective trust and procedural justice in policy identification of health impact assessment. They provide valuable insights to developing interventions to overcome barriers to the implementation and enhancement of global identification of policies. Going forward, cross-sectoral synergies, enhanced international communication and training to increase participants’ trust in the policy should be optimized to improve health impact assessment. Additional measures should be taken, such as ensuring seamless communication channels, embedding health impact assessment in administrative mechanisms, and establishing strong oversight and grievance mechanisms to improve fairness and transparency in the implementation and results of health impact assessment.

**Supplementary Information:**

The online version contains supplementary material available at 10.1186/s12961-023-01064-9.

## Background

Health in All Policies (HiAP) is a public policy development approach aimed at improving population health and health equity. It systematically considers the health consequences associated with these public policies, seeks intersectoral collaboration, and avoids policies that could have adverse effects on health. In 1999, the WHO defined health impact assessment (HIA) as “a combination of procedures, methods, and tools by which a policy, programme or project can be judged or evaluated on the basis of its potential effects on the health of a population and the distribution of those effects within the population” [1]. Health impact assessment, as a tool element of “Health in All Policies” practice, is a professional technique and effective approach for implementing “Health in All Policies”. HIA is a comprehensive tool that prioritizes health equity and enhances consideration of potential health impacts of projects, policies and plans comprehensively and transparently [2]. The tool aims to maximize health benefits and minimize potential negative impacts. Notably, HIA is extensively disseminated and practised around the world, a phenomenon that has improved the diversification of the concept and methods. HIA is applied across a wide range of areas, including transportation, economics, employment, urban and rural planning, housing, agriculture and infrastructure, among others [3–5]. Currently, many countries and regions have established a series of procedures and tools to initiate and conduct HIA, with a strong emphasis on health in decision-making [6]. This has achieved a certain degree of institutionalization of HIA, creating a permanent demand for its use.

The core objective of HIA is to enable policymakers, especially those in cross-sectoral positions, to consciously and introspectively incorporate health, health equity and determinants of health into all policies at an early stage. Scholars are currently studying the driving forces and obstacles to HIA implementation. Results from various surveys indicate that awareness or interest in health issues and social determinants of health immensely impacts HIA implementation, while lack of understanding and knowledge of HIA among decision-makers, as well as public health experts, are key obstacles to its implementation [7]. In addition, HIA emphasizes the establishment of intersectoral cooperation on a range of public policy issues, not just health department policies [1], thus involving key stakeholders, such as decision-makers, practitioners and communities, in the process of achieving coordinated action between health and non-health sectors, their roles and responsibilities, guidance capabilities, policy target groups and interdependence [8]. These stakeholders from different industries and cultural backgrounds should identify causes of health problems and the strategies to resolve them to promote policy action in public health [9]. Since the year 2000, there has been significant research on HIA. However, the majority of publications primarily focus on methodology and guidelines, comprising conceptual papers, empirical case studies, disease and epidemic investigations, health policy and planning, environment and health, socio-economic factors, health inequality, as well as health promotion and interventions. Currently, the issue of identification of the HIA system by policymakers, planners and project developers; analysis of factors influencing this identification, has not been sufficiently investigated. Hence, research that can gauge the level of policy identification by various cross-sector stakeholders and identify the factors affecting policy identification would be beneficial for advancing HIA. Such studies will enhance the sustainability and impact of HIA, and hence improve public health and health policy.

The genesis of academic inquiry into policy identification did not occur in isolation. The theoretical research on policy identification is primarily derived from Parsons’ social system theory [10], which emphasizes that “each specific action includes cognitive elements, affective elements and evaluative elements organized together.” Among these, cognitive elements refer to knowledge, namely what can be understood from the object in the context; affective elements are related to emotions, referred to as feelings by Parsons, which constitute reactions to the object; evaluative elements are associated with how the object is judged and ranked. Notably, Zhang and Tang [11] also proposed the following general formula for public policy endorsement: policy identification = trust in political authority × procedural justice × subjective evaluation of distributive justice. It points out that the acceptability of public policy is the subjective evaluation and public endorsement of the expected results of public policy implementation by the policy object and the general public. Taking the function as *Y*—policy identification; F(*X*1)—trust in political authority; F(*X*2)—procedural justice; and F(*X*3)—subjective evaluation of distributive justice, the above formula can be expressed as: *Y* = F(*X*1·*X*2·*X*3). Notably, policy identification has been widely applied in sociology [12], leadership actions [13], innovation management [14], empirical and historical research [15], economics [16], education and educational research [17], among others. However, its role in the field of health, and healthcare systems [18], particularly in HIA research, has been overlooked. Therefore, this study broadens the application scope of policy identification, fully leveraging the role of policy identification in the field of HIA, to better comprehend the challenges and driving factors in the implementation process of HIA. This contributes to offering guidance for HIA practices in various contexts.

Coincidentally, starting in May 2020, Zhejiang Province, China, has been focussing on the construction of Healthy Cities and Health-Promoting Counties (Districts), actively practising “Health in All Policies” and exploring the framework and process of HIA. The application of “Health in All Policies” extends to government departments at all levels, including institutions responsible for policy formulation and implementation, legislative bodies and administrative agencies. Therefore, this study surveyed relevant policy, planning and project developers as well as some staff, to achieve three objectives: (1) based on Parsons’ social system theory and the general formula for factors influencing public policy identification by Zhang Yu and Tang Liangfeng, to construct an initial conceptual model of the dimensions and influencing factors of policy identification status; (2) analyse the current status of policy identification to understand the level of identification, concern, participation, attitudes and other aspects among relevant individuals towards the HIA system; and (3) based on qualitative interviews, to refine and summarize the final set of factors influencing policy identification that are suitable for the HIA field. Combine this with quantitative questionnaire surveys and structural equation modelling to analyse the mechanisms of influence between these factors and policy identification. Based on this analysis, it is imperative to investigate strategies for enhancing policy identification by specific individuals in the future.

In conclusion, with the limited number of international scholars examining the current status of policy identification in HIA and the intricate interdepartmental efforts in the HIA pilot work of Zhejiang Province, gaining insights into participants’ levels of policy cognition, sentiment and evaluations becomes particularly crucial for advancing HIA. To address these research gaps, this study, based on analysing the domestic and international status of HIA practices, developed a survey questionnaire for participants in Zhejiang Province’s HIA efforts. Additionally, it constructed a theoretical framework of policy identification to explore the mechanisms of influencing factors related to policy identification. The results of this study can further illuminate the intentions of policy implementers, providing new guidance for improving the coordination and collaboration capabilities of health administration departments and optimizing resource integration and health governance levels of public policy formulation departments.

### HIA in Zhejiang province

In May 2020, Zhejiang province launched a pilot HIA programme, as part of the construction of healthy cities and counties, to explore its framework and process. The programme was implemented in 34 cities and counties, and a project-based approach was adopted to promote the establishment of the HIA system. The responsibility for HIA falls on government departments at all levels, and an HIA evaluation system was established under the leadership of the local government to ensure the implementation of HIA work. The leadership group for the construction of healthy cities at the city level, and the leadership group for the construction of healthy counties at the county level, are responsible for organizing and coordinating HIA work within their respective administrative regions. The departments responsible for economic and social development planning and policy-making, major engineering project construction or management, are responsible for conducting HIA, integrating health into planning, policies, and major engineering projects, conducting evaluations before planning and policy-making, and applying the evaluation results. To further improve the HIA mechanism, all levels of government have strengthened cooperation with universities, and research and professional institutions. In this study, we mainly focussed on public policies and major engineering projects. HIA employs a hybrid approach, integrating both qualitative and quantitative evaluation methods along with surveys. The specific implementation path consists of seven steps, namely departmental screening, formation of expert groups, selection, analysis and evaluation, reporting and recommendations, application of results, as well as monitoring and evaluation. This study conforms to the guidelines of the Zhejiang Province health impact assessment Work Manual (2022 edition), with necessary adjustments to fit the actual situation.

## Methods

### Purpose

We reviewed relevant literature and generated a conceptual HIA model for policy identification and the influencing factors. We used a combination of qualitative and quantitative methods to better understand the current level of acceptance of HIA policies among relevant personnel and further analyse the factors and mechanisms influencing policy identification.

### Conceptual framework

To identify policy identification dimensions, we chose Parsons’ social system theory [10] as the theoretical foundation. Based on this theory, we divided the policy identification dimension into cognition, sentiment and evaluation categories. In this study, policy identification in HIA specifically refers to the common acceptance and recognition of the HIA policy by policymakers, implementers and evaluators; policy cognition mainly refers to the degree of understanding, knowledge level and depth of understanding of the policy content and implementation process by HIA pilot-related personnel, whereas policy sentiment describes the attitude and concern of HIA pilot-related personnel towards the health impact assessment policy. Policy evaluation refers to the views and evaluations of HIA participants on the actual implementation process and the effect of policies.

**Fig. 1:**
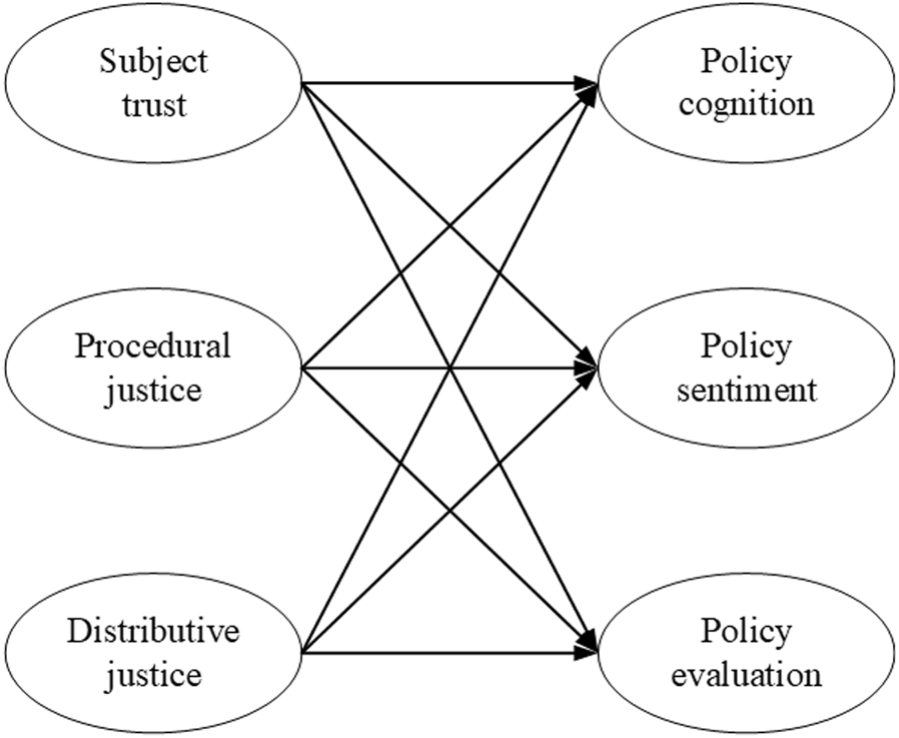
Conceptual model of policy identification and its influencing factors

Through literature review, we identified several factors that influence policy identification. Notably, Zhang and Tang [11] proposed the following general formula for determining the identification of public policy: policy identification = trust in political authority × procedural justice × subjective evaluation of distributive justice. Procedural justice significantly affects the formation of policy cognition among policy recipients [19], and the perception of procedural justice can enhance public support for policies in most public decision-making processes [20]. The public’s trust in the policy-making process also affects policy sentiment [21]. Therefore, every public policy should pay attention to the coordination of multiple interests, while policy-makers need to ensure that the process does not involve excessively polarizing attitudes to assure broad public acceptance [22]. Distributive justice, a value factor that affects “policy identification”, plays an important role in determining the behaviour choices of stakeholders, which in turn affects the effectiveness of policy identification [19]. We believe that procedural justice in HIA refers to the ability of HIA participants to conduct evaluations following the established procedures and standards, ensuring fairness and transparency during the evaluation process and ultimately obtaining objective, scientific and reliable evaluation results. HIA’s trust in the subject refers to participants’ trust in the HIA system, including evaluation institutions, experts, methods and results, among others. HIA’s distributive justice refers to fairness when evaluating specific intervention measures among different populations. Consequently, the following conceptual framework is generated (Fig. 1).

### Sample population and selection criteria

A total of 30 experts were requested to participate in one-on-one in-depth interviews. The study group comprised 15 members of the HIA system leadership group from pilot cities/counties and 15 experienced practitioners specializing in in HIA, health city construction and health promotion in counties (districts) for over 3 years. The interview questions, which were developed following a comprehensive literature review, focussed on the three dimensions of policy identification, namely policy cognition, sentiment and evaluation, as well as their influencing factors, including subject trust, procedural justice and distributive justice among others. The interviews were conducted using a clear and concise open-ended outline (Additional file 1: Appendix S1). Briefly, respondents were asked to openly share their attitudes, opinions and emotional motivations regarding public policy HIA, and also describe factors they believe affect the level of HIA identification. These views were subsequently used to identify and extract factors that influence policy identification. Informed consent was obtained from all interviewees before audio recording, and pseudonyms were used to ensure confidentiality during data transcription.

The survey targeted personnel responsible for HIA in the pilot areas in Zhejiang Province (hereinafter referred to as pilot areas). These areas mainly comprise health departments (health and health supervision departments, disease control, health education institutes, and hospitals, among others), non-health departments (transportation departments, agriculture and rural affairs bureaus, ecological environment bureaus, and water conservancy bureaus etc.), and third-party environmental assessment companies. Participants were from various regions and included people of different genders, ages, education levels, occupations and average monthly incomes. Individuals who were unwilling to participate were excluded from the study. In the survey, we employed a multi-stage stratified random sampling method. In summary, the geographical distribution of Zhejiang Province was considering when stratifying the pilot cities (counties/districts) for public policy health impact assessment, in which Hangzhou, Taizhou, Quzhou and Jiaxing were selected as representatives. A total of 40–60 individuals were selected from each city. Additionally, one to three counties/districts were randomly selected from each representative city, including Xiaoshan District, Fuyang District, Qiantang District in Hangzhou, Yuhuan City in Taizhou, Changshan County and Qujiang District in Quzhou, and Tongxiang City in Jiaxing. About 40–60 individuals were selected from each county/district for the survey, and the questionnaires were filled out and collected on-site. Ultimately, 690 questionnaires were distributed, of which 655 of the 670 that were returned were valid.

### Survey questionnaire

The survey questionnaire was designed following the Parsons’ social system theory, the identification scale [23], and semi-structured interview results. Policy identification items were designed based on Qin’s policy identification indicator system [24], while the process of measuring the influencing factors of policy identification was described in Xi’s measurement method [25] for policy identification influencing factors in combination with semi-structured interview results. Considering the Chinese assessment tools used in this, translation was carried out using the Brislin translation model and back-translation framework [26] (Additional file 1: Appendix S2). Specifically, two aspects, namely subject trust and procedural justice, were used. We also verified their reliability and validity. We incorporated all 34 items of the survey questionnaire into an exploratory factor analysis model, and then employed a principal component extraction method to extract scale components. Ultimately, five factors with eigenvalues > 1 were extracted. We also deleted 13 items with factor loadings less than 0.4, and finally remained with 21 items that covered the following five dimensions: policy cognition (four items, such as “Do you understand that health is not only the absence of disease and pain, but also the concept of physical and mental well-being and good social adaptation?”), policy sentiment (four items, such as “Do you agree that health impact assessment can involve public participation and influence decision-making?”), policy evaluation (four items, such as “What is your opinion on the level of involvement of policy stakeholders in health impact assessment?”), procedural justice (four items, such as “The establishment and implementation of health impact assessment systems have widely solicited opinions from experts and relevant representatives. Does this conform to the actual situation in your area?”), and subject trust (five items, such as “Do you believe that you can effectively consider health determinants when participating in policy, planning and projects?”).

### Data analysis

Qualitative data were obtained by conducting and recording all interviews, transcribed into Word documents, and analysed using Nvivo 12 pro software. Briefly, the data were systematically organized, and coded according to three coding stages, namely open, axial and selective coding. Next, theoretical results were continually compared and revised until a saturation was achieved. The three-level coding process not only allows analysis of the relationship between core and main category types but also reveals the relationship between various elements. The results were finally summarized in an Excel worksheet.

Quantitative data were first imputed into EpiData 3.1 software to establish a database, then checked for double entry and verification. Next, the data were subjected to descriptive statistics using SPSS 22.0 software to reveal demographic characteristics and policy identification status of HIA-related personnel. For categorical variables, frequencies and proportions are presented as mean values and standard deviations. Policy identification among individuals with different demographic characteristics were compared using one-way analysis of variance (ANOVA). The impact of procedural justice and trust among authorities on policy cognition, policy sentiment and policy evaluation was analysed using a hierarchical linear regression, while controlling for multicollinearity. Finally, the validity of the path was tested using a model constructed using Amos.

### Ethical considerations

The study was supported by Zhejiang Provincial Health Monitoring and Evaluation Center, and the study protocol was reviewed and approved by Hangzhou Normal University’s scientific research ethics committee. Before each interview, the first author introduced the research objectives and obtained verbal consent and permission to record the interview from the participants.

## Results

### Interview results

**Fig. 2:**
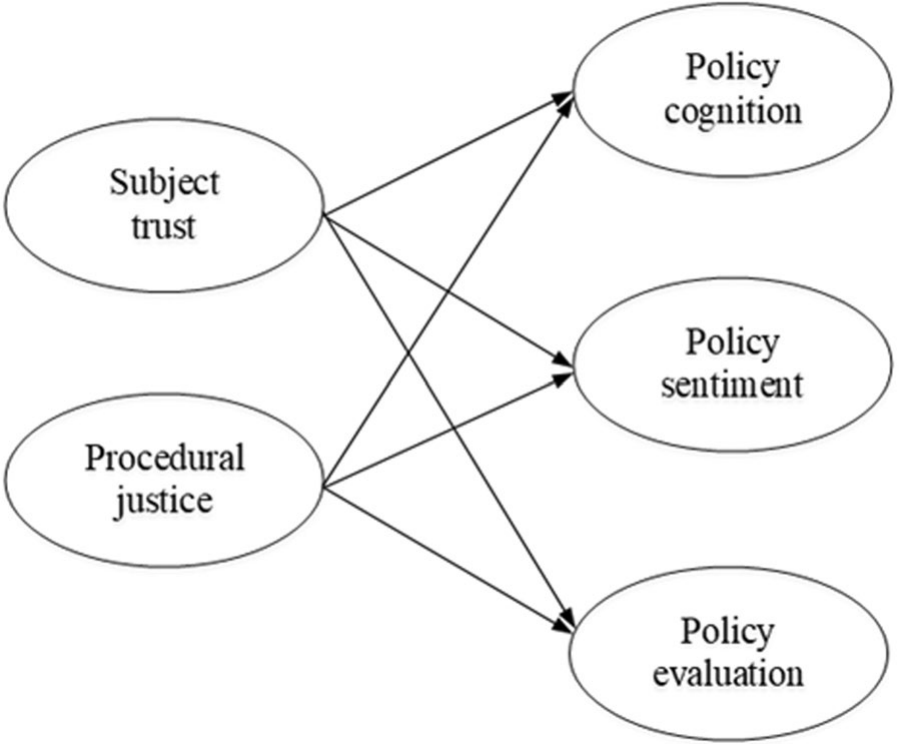
Modified conceptual model of policy identification and its influencing factors

In this study, we classified and integrated categories on the basis of the conceptual connections and logical sequences between them. This resulted in six main categories with corresponding sub- and open coding categories, shown in Additional file 1: Appendix S3. We employed a three-level coding system to extract a more comprehensive core category from the resultant main categories. Subsequently, we examined the interplay between the core category and the other primary categories. This enabled us to articulate the contextual relationships between each element, resulting in the development of a robust theoretical framework. A summary of the core categories and their relationship structures formed by selective coding is shown in Additional file 1: Appendix S4. In this study, subjective evaluations of the distributive justice mostly focus on the identification of expected policy outcomes by policy recipients. In the current pilot phase of HIA, the main focus is on researching the institutional framework, operational mechanisms, technical specifications, and evaluation processes of HIA to ensure the feasibility and operability of the HIA implementation process. Although distributive justice is an important factor in policy identification, it is mentioned less frequently in structured interviews. This is because assessing distributive justice requires more practical experience and long-term tracking of effects to draw accurate conclusions. In the pilot phase, the emphasis may be more on understanding and collecting information on the challenges of implementing HIA, operational difficulties, optimization of evaluation methods and the level of stakeholder involvement to help improve the institutional and procedural aspects of HIA. However, this does not mean that the importance of distributive justice is being ignored. In subsequent work, as HIA is further applied and its long-term effects are tracked, subjective evaluations of distributive fairness will be more widely considered and incorporated to ensure the fairness, sustainability and social justice of HIA. Therefore, this study combines the actual circumstances to modify the influencing factor model for policy identification in health impact assessment, which is defined as policy identification = subject trust × procedural justice (see the modified model in Fig. 2), with the function relationship formula expressed as *Y* = F(*X*1 × *X*2); where *Y* represents policy identification, F(*X*1) represents subject trust, and F(*X*2) represents procedural justice.

### Quantitative research results

#### Sample characteristics

The basic characteristics of the research participants are listed in Table 1. In summary, 655 individuals participated in the survey, of which 52.4% were female. The average age of the participants was 38 years, with 75.7% of them being married. With regards to employment status, a majority of the officers (85.3%) had formal employment status, with clerks (48.7%) accounting for the highest job positions. In addition, the participants had an average work experience of 11 years, with most of them (78.8%) holding a bachelor’s degree. Regarding job responsibilities, majority of participants (29.5%) were engaged in administrative management. In terms of work departments, most of the respondents (62.3%) of the officers worked in non-health departments. An analysis of the salary situation revealed that the average monthly salary for the participants was 6950 yuan. Additionally, the majority (78.8%) of individuals participated in HIA work due to higher-level requirements, and 54.8% of them reported not having any prior knowledge of HIA.

**Table 1:** Descriptive summary of respondents’ socio-demographic profiles

Demographic category	Sample size (*n* = 655)	%
Gender		
Male	312	47.6
Female	343	52.4
Marital status		
Single/divorced/widowed	159	24.3
Married	496	75.7
Mode of appointment		
Regular staff	559	85.3
The contract	96	14.7
Position		
Clerks	188	28.7
Officers	319	48.7
Deputy officers and above	148	22.6
Degree of education		
Junior college and below	58	8.9
Undergraduate course	516	78.8
Postgraduate or above	81	12.4
Job content		
Administrative management	193	29.5
Medical technology	149	22.7
Administrative management and medical technology	164	25.0
Others	149	22.7
Department of Work		
Health department	247	37.7
Non-health sector	408	62.3
Reasons for participation in HIA		
Superior requirement	516	78.8
Take one’s own initiative	84	12.8
Others	55	8.4
Have you learned about HIA?		
Yes	296	45.2
No	359	54.8

#### Preliminary analyses

We employed Cronbach’s *α* coefficient as a reliability measure for each variable, and subsequently adopted 0.7 as the threshold. The five dimensions of the policy identification questionnaire, namely trust in the subject, procedural justice, policy knowledge, policy sentiment and policy evaluation had Cronbach’s *α* coefficients of 0.912, 0.778, 0.731, 0.861 and 0.890, respectively. All these exceeded 0.7, indicating that HIA participants had relatively good internal consistency on the policy identification questionnaire. Next, we employed the composite reliability (CR) value [27] to test the internal consistency between the measurement and corresponding latent variables. Results showed that the estimated comprehensive reliability of all structures exceeded the recommended threshold of 0.7, indicating good reliability of the questionnaire (Table 2).

**Table 2:** Measurement model results

Constructs/indicators	Item loadings	*Z* value	Construct reliability	AVE
Subject trust	0.667	N/A^a^	0.895	0.633
	0.841	18.968^***^		
	0.854	19.209^***^		
	0.831	18.775^***^		
	0.914	20.218^***^		
Procedural justice	0.751	N/A^a^	0.784	0.547
	0.876	22.634^***^		
	0.865	22.371^***^		
	0.819	21.152^***^		
Policy cognition	0.697	N/A^a^	0.870	0.633
	0.593	6.290^***^		
	0.917	10.560^***^		
	0.924	10.523^***^		
Policy sentiment	0.928	N/A^a^	0.809	0.523
	0.929	32.729^***^		
	0.588	16.883^***^		
	0.602	17.453^***^		
Policy evaluation	0.718	N/A^a^	0.921	0.745
	0.807	19.722^***^		
	0.904	21.662^***^		
	0.841	20.508^***^		

^a^In AMOS, one loading has to be fixed to 1

^***^P < 0.001

AVE, average

We employed factor analysis to evaluate the structural validity of the questionnaire (Additional file 1: Appendix S2). Before conducting factor analysis, the Kaiser–Meyer–Olkin (KMO) measure and the Bartlett test of sphericity were performed to assess the variables. The KMO statistic tests the partial correlation between variables and ranges between 0 and 1, with values above 0.9 indicating that factor analysis is highly suitable. On the other hand, the Bartlett test of sphericity determines whether the variables are independent, and its significance probability (*P* value) is used to assess whether factor analysis is appropriate. In this study, the public policy health impact assessment and policy endorsement questionnaire had a KMO measure of 0.893, which was above the reference standard of 0.7. The Bartlett test of sphericity resulted in a *χ*^2^ statistic of 9380.02 with *P* < 0.001, which was statistically significant and allowed for factor analysis.

Following the two-step approach [28], we performed a confirmatory factor analysis (CFA) using the maximum likelihood estimation method test reliability and validity (convergence and discriminant validity) of the measurement model before testing our hypotheses. Next, we employed standardized factor loadings [29] and average variance extracted (AVE) [30] to evaluate convergence validity. Results showed that all standardized factor loadings were statistically significant (*P* < 0.001: Table 2). AVE reflects the variance captured by the construct related to the variance caused by measurement error, with a higher AVE value indicating a more convergent construct. A value above 0.5 is recommended, while a range of 0.36–0.5 is considered acceptable. All AVE values obtained in this study were above 0.5.

Discriminant validity analysis [31] typically involves examining the correlation or distinction between the latent characteristics of potential variables and those of other latent variables. Here, we assessed discriminant validity by comparing the square root of the AVE [31] of each dimension with the correlations between latent variables. The dimensions were deemed to have good discriminant validity if the square root of the AVE of each dimension was greater than the correlation between any two dimensions. Notably, all estimated AVE values were higher than the square correlations between structures (Table 3), indicating that each structure was statistically different from other structures.

**Table 3:** Means, standard deviations and correlations among variables

Constructs	Means	SD	1	2	3	4	5
1. Policy evaluation	3.631	0.797	**0.863**^**a**^				
2. Policy sentiment	4.137	0.664	0.525^b^	**0.723**			
3. Policy cognition	4.075	0.632	0.299	0.552	**0.812**		
4. Procedural justice	3.548	0.702	0.086	0.111	0.068	**0.686**	
5. Subject trust	3.979	0.666	0.477	0.689	0.510	0.037	**0.795**

Square root of AVE is on the diagonal (in bold). Inter-construct correlations are on the off-diagonal

^a^Average variance extracted

^b^Inter-construct squared correlations

SD, standard deviation

#### Policy identification status

Among the three policy identification dimensions, policy sentiment had the highest score (4.137 ± 0.664), followed by policy cognition (4.075 ± 0.632), with policy evaluation recording the lowest score (3.631 ± 0.797). Detailed results are shown in Table 3.

#### Single factor analysis

Next, we employed single-factor analysis to further analyse differences in policy identification and its influencing factors among the personnel involved in HIA in pilot areas of public policy. Results are shown in Additional file 1: Appendix S5. In summary, we found statistically significant differences in policy cognition among HIA participants with different appointment methods, positions, departments and whether they have been informed about HIA. Further comparison revealed that formal staff (4.10 ± 0.61) have a significantly higher (*P* < 0.05) policy cognition of health impact evaluation than contract workers (3.87 ± 0.66). Staff who hold positions of deputy director or higher (4.17 ± 0.58) had a significantly higher (*P* < 0.05) policy cognition of health impact evaluation than clerks (4.12 ± 0.61) and general staff (3.91 ± 0.66). Moreover, health department staff (4.26 ± 0.59) had a significantly higher (*P* < 0.05) policy cognition of health impact evaluation than non-health department staff (3.96 ± 0.63). Participants who had been informed about HIA had a significantly higher (*P* < 0.05) level of policy cognition regarding HIA (4.25 ± 0.61) than those who had not been informed (3.92 ± 0.60).

Furthermore, we observed that awareness of HIA and the various motivations for participating in it can impact policy sentiment. Specifically, participants with prior knowledge of HIA (4.23 ± 0.64) expressed higher levels of policy sentiment compared with those who were not aware (4.05 ± 0.67) (*P* < 0.05). Individuals actively engaged in HIA initiatives (4.35 ± 0.59) displayed significantly higher levels of policy sentiment than participants who were mandated by superiors (4.09 ± 0.67) (*P* < 0.05).

There were significant differences in the policy evaluation of HIA participants towards the HIA policy based on their marital status, job position and department. Notably, single workers rated the HIA policy (3.86 ± 0.68) significantly higher (*P* < 0.05) than married individuals (3.55 ± 0.81); HIA participants with clerks positions (3.76 ± 0.74) had a significantly higher evaluation of HIA policy than those with officers positions (3.56 ± 0.83) and those with deputy positions or above (3.60 ± 0.75) (*P* < 0.05); non-health department personnel rated the HIA policy (3.72 ± 0.73) significantly higher (*P* < 0.05) than health department personnel (3.47 ± 0.88).

#### Hierarchical linear regression

In this study, we conducted a hierarchical linear regression analysis with policy cognition, policy sentiment and policy evaluation as dependent variables. In the first layer, demographic characteristics with statistically significant differences in the univariate analysis were controlled as covariates. In the second layer, demographic characteristics and subject trust were used as independent variables, and in the third layer, demographic characteristics, subject trust and procedural justice were utilized as independent variables. The Δ*R*^2^ values were analysed to infer the impact of these variables on policy cognition, policy sentiment and policy evaluation.

Firstly, a hierarchical linear regression analysis was performed with policy cognition level as the dependent variable. The results showed that the variance inflation factors (VIF) for all independent variables were below 5, indicating no significant multicollinearity. When demographic characteristics and subject trust were included in the equation, Δ*R*^2^ became statistical significant (all *P* < 0.001). By comparing the changes in Δ*R*^2^ values, it was observed that subject trust had a larger impact on policy cognition level, explaining 24.2% of the variance. The results are shown in Additional file 1: Appendix S6. Participants who were knowledgeable about HIA had higher policy cognition scores relative to those who did not know about HIA. Additionally, subject trust had a positive effect on policy cognition among participants familiar with HIA (*β* = 0.503, *P* < 0.001).

Secondly, a hierarchical linear regression analysis was conducted with policy sentiment level as the dependent variable. The VIF values for all independent variables were below 5, indicating no significant multicollinearity. When subject trust and procedural justice were included in the equation, Δ*R*^2^ became statistically significant (all *P* < 0.01). By comparing changes in Δ*R*^2^ values, it was observed that subject trust had a larger impact on policy sentiment level, explaining 47.2% of the variance. The results are shown in Additional file 1: Appendix S7. Policy sentiment level among HIA participants increased with the scores in subject trust (*β* = 0.504, *P* < 0.001) and with higher scores in procedural justice (*β* = 0.085, *P* < 0.01).

Finally, a hierarchical linear regression analysis was performed with policy evaluation level as the dependent variable. The VIF values for all independent variables were below 5, indicating no significant multicollinearity. When demographic characteristics, subject trust and procedural justice were included in the equation, Δ*R*^2^ had statistical significance (all *P* < 0.05). By comparing the changes in Δ*R*^2^ values, it was observed that subject trust had a larger impact on policy evaluation level, explaining 20.8% of the variance. The results are shown in Additional file 1: Appendix S8. Older participants and those with higher average monthly income had lower policy evaluation scores. Policy evaluation level increased with higher scores in subject trust (*β* = 0.465, *P* < 0.001) and with higher scores in procedural justice (*β* = 0.084, *P* = 0.012).

#### Measurement model and structural model

Various fit indices were used to assess the quality of the measurement model; a *χ*^2^/degrees of freedom (df) ratio between 0 and 5 indicated a good model fit. Similarity indices such as Goodness-of-Fit Index (GFI), Adjusted Goodness-of-Fit Index (AGFI), TLI and Comparative Fit Index (CFI) with values > 0.900 also indicated a good fit. Difference indices such as root mean square error of approximation (RMSEA) and standardized root mean square residual (SRMR) with values < 0.080 indicated good model fit [32]. Model fit indices for this model (Table 4) revealed that the *χ*^2^/df ratio was 3.473, SRMR was 0.086, RMSEA was 0.061, while GFI, AGFI, Incremental Fit Index (IFI), CFI and Tucker-Lewis Index (TLI) were 0.923, 0.901, 0.952, 0.952 and 0.944, respectively. Therefore, compared with the reference values, the model had a good overall fit.

**Table 4:** Fit indices

Fit indices	Reference value	Model value
*χ*^2^/df	< 5.000	3.473
SRMR	< 0.080	0.086
RMSEA	< 0.080	0.061
GFI	> 0.900	0.923
AGFI	> 0.900	0.901
IFI	> 0.900	0.952
CFI	> 0.900	0.952
TLI	> 0.900	0.944

**Fig. 3:**
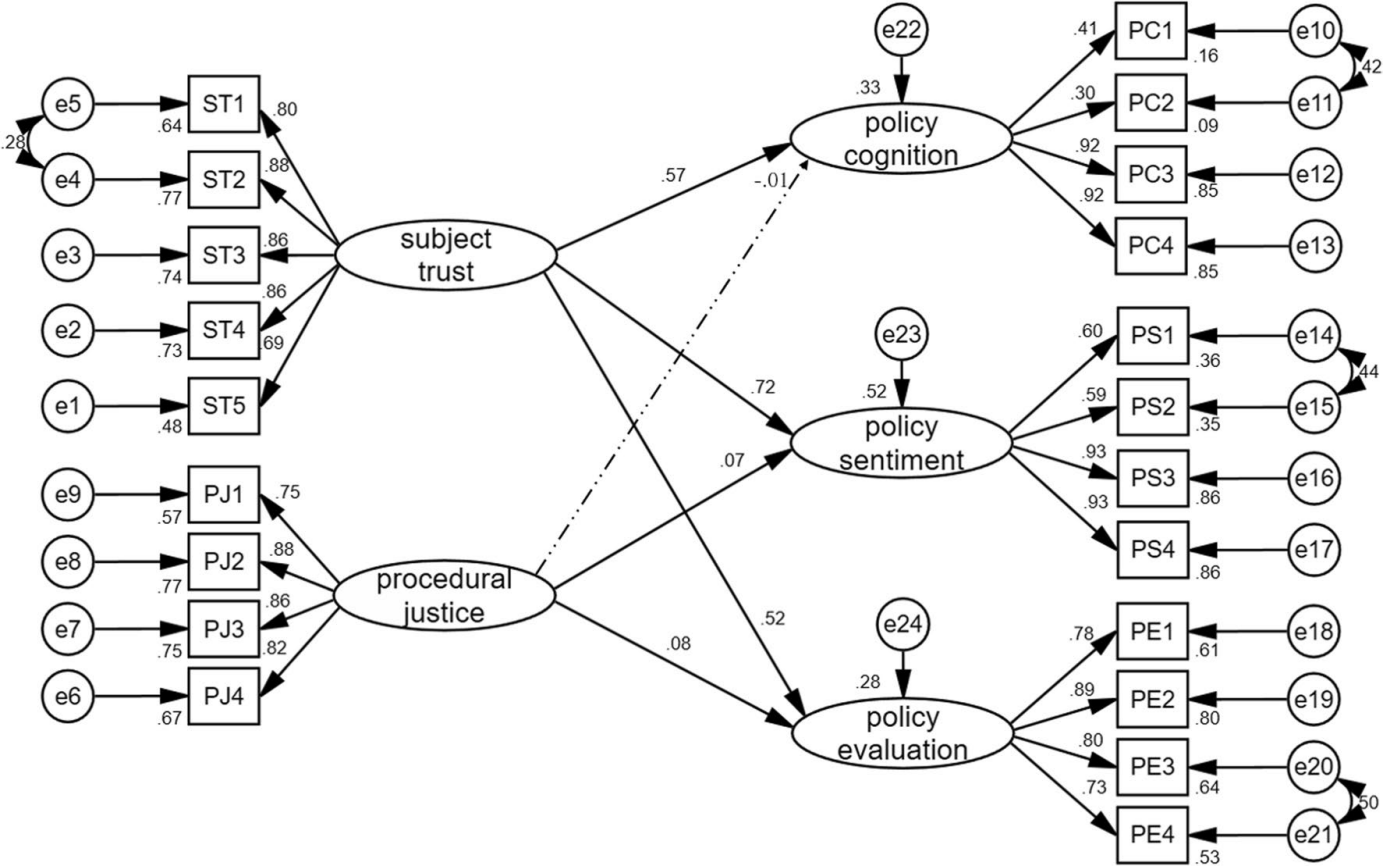
Model path diagrams and standardized estimates

In Table 5 and Fig. 3, the five hypothesized paths (H1, H2, H3, H5 and H6) in the structural model were significant in the expected direction with standardized path coefficients of 0.570, 0.716, 0.524, 0.072 and 0.078, respectively, all with*P* < 0.05, which supports the hypotheses. Only procedural justice was not significantly associated with policy cognition (*β* = −0.007, *P* > 0.05); therefore, hypothesis H4 was not supported.

**Table 5:** Estimated standardized coefficients

Hypothesized relationship	Unstandardized	SE	*Z*-value	*P*-value	Standardized	Supported?
H1: Subject trust → policy cognition	0.404	0.048	8.450	***	0.570	YES
H2: Subject trust → policy sentiment	0.625	0.050	12.435	***	0.716	YES
H3: Subject trust → policy evaluation	0.726	0.065	11.185	***	0.524	YES
H4: Procedural justice → policy cognition	− 0.004	0.020	− 0.203	0.839	− 0.007	NO
H5: Procedural justice → policy sentiment	0.042	0.019	2.236	0.025	0.072	YES
H6: Procedural justice → policy evaluation	0.072	0.036	2.015	0.044	0.078	YES

***P < 0.001

## Discussion

Building on Parsons’ social system theory and Zhang and Tang’s policy identification framework, we scrutinized the current level of policy identification among HIA participants. Two pivotal factors were identified, subject trust and procedural justice, that exert a significant influence on this identification level. The research outcomes affirm the support for all five hypotheses we put forth, furnishing valuable directional guidance for recognizing and mitigating challenges inherent in current HIA practices. The specific survey findings are outlined below.

### Analysis of the current status of HIA policy identification

In this study, HIA participants’ policy identification with HIA exhibited significant differences in policy cognition, policy sentiment and policy evaluation. Specifically, the personnel involved in HIA work expressed a positive attitude towards future HIA development; however, they lacked a deep understanding of HIA policy and were not satisfied with its implementation processes and effects. This is consistent with previous research results in China and in other countries where HIA is implemented [33–36]. In Quebec City, Canada, not all HIA participants (stakeholders) are familiar with HIA, and many people feel that their participation in HIA implementation processes is relatively low; therefore, they cannot play a greater role. They hope to learn more about the HIA processes so that they can better use it in different situations and benefit.

We analysed the differences in policy identification among participants and postulated that they could be attributed to the following reasons. With rapid development of the society and economy, people are more concerned about health than ever before, and more discussions are being held on how to live a happy and healthy life. As people become increasingly worried about their health, this has sparked the interest and motivation of stakeholders to perform HIA [1]. Moreover, as governments, health organizations and the public recognize the need for more comprehensive assessment of health impacts, people have renewed their interests in health impact assessment methods [37]. However, HIA overlaps with other sectors such as environmental, cultural and social impact assessments [38] and is inherently interdisciplinary, where knowledge is not only generated through the collaboration of experts from various disciplines, but also through interactions with local stakeholders and citizens [39]. This dynamic influences both the health sector and non-health sectors in their comprehension and implementation of HIA policies. Certain educational institutions have a unique role to play in HIA implementation and can narrow the gap between HIA practice and knowledge among health and non-health sector personnel [40]. Therefore, development of HIA teaching and training products will improve the cognitive levels of HIA participants [7]. There have been advances in HIA; however, due to financial and manpower constraints, as well as imperfect participation mechanisms at the local level, the actual implementation has not met people’s expectations [41].

### Analysis of factors influencing HIA policy identification

Regarding the factors influencing HIA policy identification, after controlling for demographic variables, our hierarchical linear regression results align with the structural equation model path analysis results.

#### Policy cognition

Our results indicate that participants who had prior knowledge of HIA exhibited higher levels of cognition compared with those who had no knowledge about HIA, which is consistent with the results of Li’s study [42]. Currently, over half of the HIA participants (54.8%) reported that they never learned about HIA, indicating that publicity about HIA is not widespread. Furthermore, subject trust has a significant positive impact on participants’ policy cognition level, reflecting the prevalent notion that fundamental cognitive processes depend on trust (Ploywarin [43], Mayo [44]). In particular, if a majority of HIA participants hold the belief that HIA policies are carefully formulated with due consideration for public interests and the promotion of public health, this conviction may increase their appreciation for HIA policies. This can motivate them to actively pursue information pertaining to HIA, subsequently influencing their levels of cognition and comprehension regarding HIA. Conversely, if HIA participants harbour doubts about HIA policies, they may not actively seek out relevant information. This sceptical mindset might lead to reduced interest in policies, diminishing their motivation to delve into policy details, thereby limiting their interaction with policies and their comprehensive understanding and awareness of them. It is worth noting that procedural justice has no significant effect on policy cognition, which seems to be different from Bai et al. [45], who revealed that procedural justice can promote knowledge acquisition to improve cognition. The disparity arises from the distinct research contexts of the two studies. Bai et al. explored the functioning of procedural justice within business alliances. In contrast, our study focusses on a provincial pilot project for HIA, examining how procedural justice impacts the cognitive processes of policy participants. In reality, the survey of policy cognition among HIA participants places greater emphasis on aspects such as policy objectives, planning and scope, with relatively less focus on procedural details of policy formulation and implementation. This situation may be partly because policy cognition is influenced not only by the fairness of policy procedures but also by individual backgrounds, experiences, beliefs and other factors. In this context, HIA participants are more inclined to focus on how policies meet societal needs and address issues. They may be more concerned about the practical impact of policies rather than just the details within the policy procedures.

#### Policy sentiment

The research results indicate that subject trust positively influences policy sentiment. This finding aligns with the studies by Rodriguez-Sanchez et al. [46] and Yuan et al. [47], where they found that the general trust of the public in the government and its policies affects people’s emotional response to policies. This emotional response, in turn, directly or indirectly impacts policy acceptance through anticipated costs and benefits. Establishment of a health impact assessment system covering the provincial level is a new initiative in China. When HIA participants trust the HIA policies, they anticipate that the government will implement the “health in all policies” strategy through HIA, promoting democracy and public health. This trust also instills confidence in HIA participants regarding engaging in HIA work. Furthermore, we found that procedural justice has a positive impact on policy sentiment. This research result is consistent with the studies by Jang et al. [48], Edward et al. [49] and Wu et al. [50]. Specifically, procedural injustice can trigger emotional responses such as anger, frustration or fear. Implementing procedural justice in policy execution can reduce anger and frustration among relevant individuals, thereby enhancing policy compliance.

#### Policy evaluation

Research has revealed a negative correlation between age and monthly income with policy evaluation, which is in partial agreement with the perspective presented by Xiao et al. [51]. Considering the practical circumstances, it is reasonable to speculate that, due to the government’s close attention and emphasis on the pilot work of HIA, participants with relatively higher age and income levels typically bear greater work pressure and responsibilities. They exhibit increased concern for the actual effects and long-term outcomes of policies. When the implementation effects of HIA are not sufficiently prominent or require an extended period to fully manifest, they may exhibit a more cautious evaluation of policies. This cautious evaluation might stem from their higher expectations regarding policy impact and a more stringent standard for policy success. Subject trust significantly influences policy evaluation. Albrecht [52] and Wals [53] reported a positive correlation between increased public trust in the government and heightened policy support. Ma [54] suggests that to achieve better policy evaluation, the government must prioritize establishing trust in decision-making execution, thereby enhancing public support and approval of policies. When HIA participants have a high level of trust in the government and its formulated HIA policies, they are more inclined to accept the objectives and principles of the policy, believing it is designed for the benefit of the public. This trust leads to a more active and open evaluation of policy implementation, not solely from a personal or specific interest standpoint. Conversely, if different stakeholders have varying interpretations of the objectives HIA should achieve, reaching a consensus on the anticipated effects of HIA implementation becomes challenging. This divergence may influence trust, potentially leading to negative evaluations. In addition, procedural justice has a positive impact on policy evaluation. This aligns with the research findings of Martin et al. [55] and Liu B et al. [56], who discovered a positive correlation between procedural justice and policy evaluation. The survey results further emphasize that the procedural fairness of policies makes participants more readily accept policy outcomes and raises fewer doubts.

### Measures to enhance HIA policy identification

Enhancing the trust of HIA participants in HIA policy helps enhance their identification of the policy. Trust is an essential component of effective governance in all major areas of public policy and is crucial for enhancing policy legitimacy [57]. Thus, when conducting HIA activities, a cross-sectoral collaborative mechanism should be established firstly. Through the integration of independent organizational resources, facilitation of a coordinated framework, and allocation of goals and responsibilities to different government departments, a trust-based multi-sectoral collaboration can be established. This ensures that HIA participants from various positions and departments fully understand policy-related information. Together, they can collectively study and review major issues in HIA implementation, thereby enhancing the acknowledgment, respect and trust of HIA policy among participants. Secondly, to strengthen HIA practice and improve the quality of assessments, it is vital to organize training, international exchanges, workshops and seminars. Indeed, while HIA is widely used in some regions, its education and training are relatively limited globally [58]. Therefore, it is urgent to learn and draw from international advanced assessment methods and experiences. This will enhance the objective analysis, evaluation and discussion of HIA by multi-disciplinary experts, elevate the assessment quality and scientific rigor, thereby strengthening the trust in HIA policy among participants. It ensures they can effectively engage in the HIA process and deeply understand and support the HIA policy.

Promoting the procedural construction of HIA policy and enhancing the sense of procedural justice among HIA participants will increase their policy identification. First, the government should establish effective communication channels. It is crucial to not only incorporate the perspectives of vulnerable groups and citizens [37], but also to allow experts from diverse fields to engage in health impact assessments and express their viewpoints openly. Additionally, as our country endeavours to establish a comprehensive HIA system, it is worth noting that HIA is presently considered a “selective action” conducted prior to the government’s formulation of public policies, plans and projects. There is a need to further integrate HIA into the administrative framework. The government should establish a corresponding framework specifying when and to what extent HIA is required [3] and provide information on all stages of HIA implementation, ensuring transparency in supervision [59]. This will improve their perceptions of fairness and justice in the policy design and implementation process, thereby increasing policy recognition for HIA. Lastly, to ensure a fair and transparent assessment process and results, the government should establish supervisory and grievance mechanisms. This allows HIA participants to provide feedback and complaints based on the decision-making process and assessment outcomes. In turn, decision-making departments can continuously refine policies to ensure their effectiveness and sustainability. This continuous monitoring mechanism fully utilizes the key role of HIA participants, urging the government to seriously respond to assessment outcomes and feedback and take necessary measures to refine policies, protecting the public interest. By implementing the aforementioned measures, we can foster an environment of procedural justice, where individuals engaged in HIA operate within a fair, transparent and well-organized framework. This not only upholds the integrity and trustworthiness of the decision-making process but also amplifies participants’ endorsement of HIA policies, ultimately advancing the effectiveness and sustainability of HIA practice.

## Limitations

This study has several limitations. First, since the data collected in this study was cross-sectional, factors that may impact policy identification were not fully considered. Second, as HIA needs to consider economic, technological and other social factors, too strict quantitative analyses may decrease the acceptability of HIA policies. Third, the strategy of “Health in All Policies” aims to increase the responsibility of policy makers at all levels towards health, prompting various departments to more consciously and sensitively shoulder health responsibilities. At present, HIA is in the pilot stage in China, and this phase focusses more on whether HIA can effectively promote progress. Consequently, the main focus of the study is on relevant policy, planning and project developers as well as some staff. While HIA participants are essential components of policy implementation, the ultimate target of policy recognition is the public. Gaining a deeper understanding of public opinions would be beneficial for advancing HIA and democratization. In subsequent research, it is advisable to include the public in the scope of the study, comprehend their perspectives on HIA policies, and attitudes. This would further address the limitations of the participant-centric viewpoint, providing a more comprehensive and objective data support that aligns with public needs and expectations, enhancing policy acceptance and implementation effectiveness. Finally, the relationship between policy identification, subject trust and procedural justice is a complex dynamic process. These concepts have interactive effects and mediating factors that require further research and understanding. Therefore, exploring the mediating factors between policy identification and subject trust, and procedural justice will be an important direction for future research. These studies will provide policymakers with a deeper understanding, enabling them to design more attractive and credible policies, and promote public acceptance and support for policies.

## Conclusions

Globally, HIA is increasingly being applied in various countries; however, its practice is limited by various challenges and difficulties. To enhance the practical value of HIA, efforts must be made to address the problems encountered in the application process. Policy identification is crucial for policy implementation and execution. Therefore, based on the perspective of policy identification, this study has constructed a theoretical framework for HIA policy identification and explored in depth the impact mechanism of procedural justice and subject trust on policy identification, providing improvement for the field of policy identification and HIA research. We conducted a survey within the scope of Zhejiang Province to investigate the HIA participants’ identification with HIA policies. Although the level of identification with HIA policies among participants was relatively high, there is still room for improvement. Furthermore, we emphasized the positive influence of subject trust and procedural justice on policy identification, and these research findings are supported by empirical evidence from the process of HIA institutional construction in Zhejiang Province. Therefore, we further put forward improvement suggestions, such as establishing a cross-departmental collaboration platform for HIA, conducting training and exchanges, facilitating channels for opinion communication, disclosing process information, and establishing supervision and appeal mechanisms, to enhance subject trust and procedural justice, thereby enhancing participants’ identification with policies and promoting the smooth implementation and optimization of HIA. This not only provides action guidance for Zhejiang Province and other HIA pilot regions, but also contributes valuable experience to the global promotion of HIA.

### Supplementary Information

**Additional file 1.** Supplementary material. **Appendix 1** Interview Guide. **Appendix 2** Questionnaire. **Appendix 3** Main category formed by spindle encoding. **Appendix 4** Core categories and their relational structure as formed by selective coding. **Appendix 5** Univariate analysis. **Appendix 6** Stratified regression analysis of policy cognition level. **Appendix 7** Stratified regression analysis of policy sentiment level. **Appendix 8** Stratified regression analysis of policy evaluation level.

## Data Availability

All data were treated as confidential and not publicly available but could be disclosed through the correspondence author on a reasonable request.

## Abbreviation

HIA: Health impact assessment
