# Supplementary material for: Current status and influencing factors of policy identification in health impact assessment: a case study of Zhejiang Province
Source: Health Res Policy Syst. 2023 Nov 6;21:118. doi: 10.1186/s12961-023-01064-9 (PMC10626660; doi:10.1186/s12961-023-01064-9)
Supplement: Supplementary file 1 — Additional file 1. Supplementary material. Appendix 1 Interview Guide. Appendix 2 Questionnaire. Appendix 3 Main category formed by spindle encoding. Appendix 4 Core categories and their relational structure as formed by selective coding. Appendix 5 Univariate analysis. Appendix 6 Stratified regression analysis of policy cognition level. Appendix 7 Stratified regression analysis of policy sentiment level. Appendix 8 Stratified regression analysis of policy evaluation level. [file 12961_2023_1064_MOESM1_ESM.docx]

**Additional file**

**Appendix S1 Interview Guide**

| **No.** | **Interview Questions** |
| --- | --- |
| 1 | Have you ever conducted Health Impact Assessment (HIA) in your daily work? |
| 2 | Under what circumstances would you choose to conduct or not conduct HIA? |
| 3 | Has your unit or department promoted HIA-related knowledge? |
| 4 | Are you familiar with HIA policies, such as classification criteria for health factors, and health equity among others? |
| 5 | What is your perspective on HIA policies? |
| 6 | To the best of your knowledge, how do other individuals involved in HIA work perceive HIA? Have they praised or complained about HIA? |
| 7 | To the best of your knowledge, what is the level of identification of HIA policies in your department or organization? |
| 8 | To the best of your knowledge, does the HIA system in your area reflect opinions and suggestions through continuous monitoring and evaluation? |
| 9 | Do you believe that inclusion of HIA in the whole policy formulation process by various departments can effectively exert the guiding role of public policy on public health and effectively protect the health rights and interests of urban and rural residents? |
| 10 | Do you feel that your level of involvement in HIA work has been matched by a corresponding level of return? |
| 11 | What factors do you think influence the identification level of HIA policies? |
| 12 | What do you think is the most critical aspect of HIA during the execution process? Please explain. |
| 13 | In your opinion, what parts of HIA deviates from the policy text in implementation processes? |
| 14 | What aspects of the HIA system do you think should be strengthened? Please explain. |
| 15 | Can you provide some suggestions and opinions on the HIA policy? Please explain. |

**Appendix S2** **Questionnaire**

| **Survey item** | **Likert item** | | | | |
| --- | --- | --- | --- | --- | --- |
| 1.Are you familiar with the concept that health is not just the absence of disease and pain, but also encompasses physical and mental well-being, as well as a good social adaptation state? | 1 | 2 | 3 | 4 | 5 |
| 2.Are you familiar with the concept of Health Impact Assessment, which is a series of procedures, methods, and tools used to systematically assess the potential impact of policies, plans, and projects (usually involving multiple departments or cross-departments) on the health of populations, as well as the distribution of these impacts among populations? | 1 | 2 | 3 | 4 | 5 |
| 3. Do you agree that the Health Impact Assessment expert group should be familiar with the technical processes of Health Impact Assessment before conducting an evaluation? | 1 | 2 | 3 | 4 | 5 |
| 4. Do you agree that the Health Impact Assessment expert group should be familiar with definitions and explanations of health, health equity, and health determinants in analysis and evaluation table of Health Impact Assessment before conducting an evaluation? | 1 | 2 | 3 | 4 | 5 |
| 5. On a scale of 1 to 5, how would you rate the current performance of the Disease Control and Prevention Center in terms of checking the rationality of the Health Impact Assessment process, the suitability of the methods, the comprehensiveness of the health issues involved in Health Impact Assessment, and the overall monitoring and feedback of Health Impact Assessment? | 1 | 2 | 3 | 4 | 5 |
| 6. How would you rate the current situation of the Health Bureau in formulating the implementation measures of Health Impact Assessment, organizing and coordinating, supervising and inspecting the Health Impact Assessment work in its jurisdiction? | 1 | 2 | 3 | 4 | 5 |
| 7. How would you rate the current level of participation of policy stakeholders in Health Impact Assessment? | 1 | 2 | 3 | 4 | 5 |
| 8. On a scale of 1 to 5, how would you rate the current level of implementation of "health in all policies"? | 1 | 2 | 3 | 4 | 5 |
| 9. On a scale of 1 to 5, do you agree that Health Impact Assessment can involve public participation in Health Impact Assessment and influence decision-making? | 1 | 2 | 3 | 4 | 5 |
| 10. Do you agree that Health Impact Assessment can assess the short-term and long-term effects of each proposal and provide them to decision-makers in a timely manner? | 1 | 2 | 3 | 4 | 5 |
| 11. Do you agree that establishment and implementation of the Health Impact Assessment system by widely soliciting the opinions of experts and representatives of relevant groups is in line with the actual situation in your region? | 1 | 2 | 3 | 4 | 5 |
| 12. Based on the actual situation in your region, is the information in the process of implementing Health Impact Assessment open, fair, transparent, and equitable? | 1 | 2 | 3 | 4 | 5 |
| 13. Does the preparation of policies with Health Impact Assessment have standardized procedures and processes in your region? | 1 | 2 | 3 | 4 | 5 |
| 14. Is Health Impact Assessment open, transparent, and does it rigorously utilize evidence and methods from different disciplines to assess impacts and provide recommendations in line with the actual situation in your region? | 1 | 2 | 3 | 4 | 5 |
| 15. Do you actively follow the dynamics of China's policies that are related to Health Impact Assessment? | 1 | 2 | 3 | 4 | 5 |
| 16. Do you actively follow the development of evaluation methods for Health Impact Assessment? | 1 | 2 | 3 | 4 | 5 |
| 17. Do you have confidence in being familiar with administrative procedures for government policy making? | 1 | 2 | 3 | 4 | 5 |
| 18. Do you have confidence in mastering the process and steps of Health Impact Assessment? | 1 | 2 | 3 | 4 | 5 |
| 19. Can you correctly understand and comprehend health determinants? | 1 | 2 | 3 | 4 | 5 |
| 20. Can you effectively consider health determinants when participating in formulation of policies, plans, and projects? | 1 | 2 | 3 | 4 | 5 |
| 21. Are you willing to participate in groundbreaking work related to Health Impact Assessment? | 1 | 2 | 3 | 4 | 5 |

**Appendix S3 Main category formed by spindle encoding**

| **Main category** | **Corresponding category** | **Connotation of category relationship** |
| --- | --- | --- |
| Formation of the consultation process | Incorporation of opinions | The degree to which opinions of diverse policy stakeholders, such as domain experts, professionals, social groups, and individual citizens are taken into account will impact the solicitation process of public policy health impact assessment. |
|  | Scientific evaluation | The strength of expert technical guidance and level of expert database construction will impact the solicitation process of public policy health impact assessment. |
| Formation of the workflow process | Information disclosure | The scope and intensity of policy promotion and interpretation as well as effective transparency of policy implementation details will impact the process and procedures of public policy health impact assessment. |
|  | Standardized procedures | Standardization of the implementation process and scope procedures of health impact assessment will impact the process and procedures of public policy health impact assessment. |
| Formation of evaluation feedback process | Monitoring feedback | Standardization of assessment mechanisms and the legal processes, as well as level of legal effectiveness will impact the evaluation and feedback procedures of public policy health impact assessment. |
| Formation of trust in policy participation | Policy effectiveness | The level of government attention, support, and administrative promotion of health impact assessment work, the coordinating and guiding role of the health department in pilot work of health impact assessment, and effectiveness of cross-departmental collaboration will impact policy participation trust of public policy health impact assessment. |
|  | Consideration of concerns | Adequate investment of human, financial, and material resources, policy guarantees for expanding personnel participation, and social optimization as well as adjustment of health impact assessment work will impact policy participation trust of public policy health impact assessment. |
| Formation of trust in policy implementation | Administrative procedures | Simplification and conciseness of the evaluation process and prioritization of evaluation steps will impact policy implementation trust of public policy health impact assessment. |
|  | Evaluation methods | Standardization and rigor of the evaluation process and objectivity as well as professionalism of expert evaluation opinions will impact policy implementation trust of public policy health impact assessment. |
| Formation of trust in policy making | Integration into policy-making | Organic integration of health impact assessment work content with work priorities of various departments and the specific implementation path of top-level design will impact policy formulation trust of public policy health impact assessment. |
|  | Deep cooperation | Substantive refinement and exploration of work templates and case models will impact policy formulation trust of public policy health impact assessment. |

**Appendix S4 Core categories and their relational structure as formed by selective coding**

| **Core category** | **Main category** | **Interpretation of category relationship structure** |
| --- | --- | --- |
| Subject trust | Policy participation trust | Policy participation trust affects the sense of identification of government officials with trust of the subject. |
|  | Policy implementation trust | Policy implementation trust affects the sense of identification of government officials with trust of the subject. |
|  | Policy formulation trust | Policy formulation trust affects the sense of identification of government officials with trust of the subject. |
| Procedural justice | Consultation procedures | Solicitation of opinion procedures affects the sense of procedural fairness for government administrative staff. |
|  | Process procedures | Process procedures affects the sence of procedural fairness for government administrative staff. |
|  | Evaluation of feedback procedures | Process procedures affects the sense of procedural fairness for government administrative staff. |

**Appendix S5 Univariate analysis**

| **Variable** | **Demographic Category** | **Policy**  **cognition** | **Policy**  **sentiment** | **Policy evaluation** |
| --- | --- | --- | --- | --- |
|  |  | **M±SD** | **M±SD** | **M±SD** |
| **Gender** | Male | 4.07±0.62 | 4.12±0.64 | 3.60±0.80 |
|  | Female | 4.07±0.64 | 4.14±0.67 | 3.65±0.79 |
| t(*p*) |  | -0.04（0.966） | -0.47（0.638） | -0.79（0.427） |
| **Marital status** | Single/Divorced/Widowed | 4.05±0.61 | 4.15±0.65 | 3.86±0.68 |
|  | Married | 4.08±0.63 | 4.13±0.66 | 3.55±0.81 |
| t(*p*) |  | -0.45（0.651） | 0.47（0.635） | **4.79（＜0.001）** |
| **Mode of appointment** | Regular staff | 4.10±0.61 | 4.15±0.65 | 3.61±0.80 |
|  | The contract | 3.87±0.66 | 4.02±0.72 | 3.72±0.72 |
| *t(p)* |  | **3.42（0.001）** | 1.73（0.083） | -1.30（0.191） |
| **position** | Clerks | 3.91±0.66 | 4.07±0.70 | 3.76±0.74 |
|  | Officers | 4.12±0.61 | 4.14±0.66 | 3.56±0.83 |
|  | Deputy Officers and above | 4.17±0.58 | 4.19±0.58 | 3.60±0.75 |
| F*(p)* |  | **8.87（＜0.001）** | 1.55（0.213） | **3.62（0.027）** |
| **Degree of education** | Undergraduate and below | 3.90±0.67 | 4.00±0.68 | 3.68±0.77 |
|  | Postgraduate or above | 4.07±0.63 | 4.16±0.66 | 3.64±0.80 |
| *t (p)* |  | -1.90（0.057） | -1.70（0.089） | 0.34（0.731） |
| **Department of Work** | Health department | 4.26±0.59 | 4.16±0.67 | 3.47±0.88 |
|  | Non-health sector | 3.96±0.63 | 4.12±0.66 | 3.72±0.73 |
| *t (p)* |  | **6.13(<0.001)** | 0.71(0.474) | **-3.78(<0.001)** |
| **Reasons for participation in HIA** | Superior requirement | 4.06±0.63 | 4.09±0.67 | 3.59±0.79 |
|  | Take one's own initiative | 4.16±0.61 | 4.35±0.59 | 3.82±0.81 |
|  | others | 4.01±0.59 | 4.17±0.62 | 3.63±0.76 |
| *F(p)* |  | 1.06（0.344） | **5.44（0.005）** | 2.82（0.060） |
| **Have you learned about HIA?** | YES | 4.25±0.61 | 4.23±0.64 | 3.65±0.88 |
|  | NO | 3.92±0.60 | 4.05±0.67 | 3.61±0.71 |
| *t(p)* |  | **6.71（＜0.001）** | **3.57（＜0.001）** | 0.60（0.547） |

**Appendix S6 Stratified regression analysis of policy cognition level**

| **Variable** | | | **The 1st level** | **The 2nd level** | **The 3rd level** |
| --- | --- | --- | --- | --- | --- |
|  |  |  | **Standard *Beta*** | **Standard *Beta*** | **Standard *Beta*** |
| **Age** |  | 0.008 | 0.041 | 0.036 |  |
| **Years of work experience** |  | -0.012 | 0.007 | 0.010 |  |
| **Employment mode**  (reference group = Formal staff) | The contract | -0.054 | -0.054 | -0.060 |  |
| **Position**  (reference group=Clerks) | Officers | 0.097 | 0.081 | 0.078 |  |
|  | Deputy Officers and above | 0.114* | 0.068 | 0.064 |  |
| **Department of Work**  (Reference Group = Health Department) | Non-health sector | -0.034 | -0.031 | -0.039 |  |
| **Average monthly salary** |  | 0.033 | 0.007 | 0.005 |  |
| **Have you learned about HIA?** （Reference Group =YES） | NO | -0.237*** | -0.155*** | -0.154*** |  |
| **Subject trust** |  | / | 0.504*** | 0.503*** |  |
| **Procedural justice** |  | / | / | 0.056 |  |
| R^2^ |  | 0.099 | 0.342 | 0.345 |  |
| F |  | 4.120*** | 18.325*** | 17.565*** |  |
| ΔR^2^ |  | 0.099 | 0.242 | 0.003 |  |
| ΔF |  | 4.120*** | 234.181*** | 2.896 |  |
| VIFmax |  | 3.989 | 3.989 | 4.020 |  |

Note：*=*P*<0.05；**=*P*<0.01；***=*P*<0.001

**Appendix S7 Stratified regression analysis of policy sentiment level**

| **Variable** | | | **The 1st level** | **The 2nd level** | **The 3rd level** |
| --- | --- | --- | --- | --- | --- |
|  |  |  | **Standard *Beta*** | **Standard *Beta*** | **Standard *Beta*** |
| **Age** |  | 0.024 | 0.066 | 0.060 |  |
| **Years of work experience** |  | -0.067 | -0.040 | -0.036 |  |
| **Average monthly salary** |  | 0.021 | -0.013 | -0.017 |  |
| **Reasons for participation in HIA**  （Reference Group = Superior requirement） | Take one's own initiative | 0.080* | 0.050 | 0.050 |  |
| **Have you learned about HIA?** （Reference Group =YES） | NO | -0.138** | -0.023 | -0.022 |  |
| **Subject trust** |  | / | 0.703*** | 0.702*** |  |
| **Procedural justice** |  | / | / | 0.085** |  |
| R^2^ |  | 0.03 | 0.503 | 0.509 |  |
| F |  | 1.171 | 35.697*** | 34.695*** |  |
| ΔR^2^ |  | 0.030 | 0.472 | 0.007 |  |
| ΔF |  | 1.171 | 603.789*** | 8.791** |  |
| VIFmax |  | 3.989 | 3.989 | 4.020 |  |

Note：*=*P*<0.05；**=*P*<0.01；***=*P*<0.001

**Appendix S8 Stratified regression analysis of policy evaluation level**

| **Variable** | | | **The 1st level** | **The 2nd level** | **The 3rd level** |
| --- | --- | --- | --- | --- | --- |
|  |  |  | **Standard *Beta*** | **Standard *Beta*** | **Standard *Beta*** |
| **Age** |  | -0.126** | -0.085* | -0.090* |  |
| **Marital status**  (reference group = Single/Divorced/Widowed) | Married | -0.107 | -0.046 | -0.037 |  |
| **Position**  (reference group=Clerks) | Officers | -0.067 | -0.082 | -0.087 |  |
|  | Deputy Officers and above | 0.020 | -0.023 | -0.028 |  |
| **Years of work experience** |  | -0.024 | -0.006 | -0.004 |  |
| **Department of Work**  (Reference Group = Health Department) | Non-health sector | 0.042 | 0.046 | 0.034 |  |
| **Average monthly salary** |  | -0.105* | -0.128*** | --0.132*** |  |
| **Subject trust** |  |  | 0.466*** | 0.465*** |  |
| **Procedural justice** |  |  | / | 0.084* |  |
| R^2^ |  |  | 0.281 | 0.287 |  |
| F |  |  | 13.797*** | 13.482*** |  |
| ΔR^2^ |  |  | 0.208 | 0.007 |  |
| ΔF |  |  | 183.532*** | 5.895* |  |
| VIFmax |  |  | 3.989 | 4.020 |  |

Note：*=*P*<0.05；**=*P*<0.01；***=*P*<0.001
